# Intractable Hiccups in a Patient With End-Stage Renal Disease: A Five-Month Ordeal Resolved Through Hemodialysis

**DOI:** 10.7759/cureus.61725

**Published:** 2024-06-05

**Authors:** Piercarlo Minoretti

**Affiliations:** 1 Occupational Health, Studio Minoretti, Oggiono, ITA

**Keywords:** chronic renal failure, chlorpromazine, baclofen, hemodialysis, covid-19, intractable hiccups

## Abstract

Hiccups, a common and usually self-limiting condition, are caused by involuntary, spasmodic contractions of the diaphragm and intercostal muscles, followed by the sudden closure of the glottis. While most cases resolve spontaneously, persistent hiccups (lasting 48 hours to one month) and intractable hiccups (lasting more than one month) require medical attention. Intractable hiccups, although rare, can significantly impair a patient's quality of life. The etiology of intractable hiccups is diverse, but they are often associated with serious underlying medical conditions, such as severe renal dysfunction and uremia. We present the case of a 72-year-old male patient with stage IV chronic kidney disease (CKD) who developed intractable, violent hiccups following a mild COVID-19 infection. Despite treatment attempts with chlorpromazine and baclofen, the hiccups persisted for five months and only resolved after the initiation of hemodialysis. Interestingly, the patient's renal function deteriorated significantly during the period of hiccup persistence, suggesting a possible link between the hiccups and the progression of CKD, likely exacerbated by COVID-19. This case highlights the challenges of managing intractable hiccups in patients with advanced CKD and emphasizes the importance of addressing underlying metabolic derangements in such complex clinical scenarios. Moreover, it contributes to the growing evidence supporting the role of dialysis in resolving intractable hiccups associated with severe renal dysfunction.

## Introduction

Hiccups are a common and typically self-limiting condition characterized by involuntary, spasmodic contractions of the diaphragm and intercostal muscles, followed by the sudden closure of the glottis [1]. In most instances, hiccups are benign and resolve spontaneously within a few minutes to a few hours. However, in rare cases, hiccups may persist for longer durations [2]. Persistent hiccups are characterized by bouts lasting longer than 48 hours but less than one month, whereas intractable hiccups refer to episodes persisting for more than one month [3,4]. Intractable hiccups can lead to complications such as exhaustion, feeding difficulties, disrupted sleep patterns, and significant impairment in a patient's quality of life [5]. Although potentially idiopathic or drug-induced in certain cases, intractable hiccups necessitate medical attention as they are frequently linked to serious underlying medical conditions, including brain tumors, strokes, severe gastrointestinal diseases, and toxic-metabolic states, including severe renal dysfunction and/or uremia [6].

At present, there is a paucity of robust evidence to guide treatment recommendations for intractable hiccups [4]. Nevertheless, the medical literature documents the efficacious use of various pharmacological interventions in the management of persistent and refractory hiccups. These therapeutic agents include chlorpromazine, baclofen, gabapentin, amitriptyline, metoclopramide, nifedipine, midazolam, nimodipine, haloperidol, orphenadrine, and valproic acid [4]. While chlorpromazine remains the sole medication approved by the United States Food and Drug Administration (FDA) specifically for the treatment of hiccups, baclofen and gabapentin are also frequently regarded as first-line therapies in clinical practice [1,4].

In this report, we present the case of a patient with stage IV chronic kidney disease (CKD) who developed intractable, violent hiccups following a mild COVID-19 infection. The onset of hiccups coincided with a rapid deterioration in kidney function, most likely driven by COVID-19, and proved refractory to treatment attempts with chlorpromazine and baclofen. After persisting for a total of five months, the intractable hiccups, which had led to significant patient prostration, resolved after the initiation of hemodialysis.

## Case presentation

A 72-year-old male patient with a history of chronic atrial fibrillation managed with oral anticoagulation therapy, hiatal hernia, and stage IV CKD according to the Kidney Disease Outcomes Quality Initiative (KDOQI) guidelines, under conservative management (Table 1) and regular surveillance through three-month outpatient visits and blood tests, contracted mild COVID-19 in March 2021.

**Table 1:** Pre-dialysis medical treatment

Drug	Dosage
Bisoprolol	1.25 mg, twice per day
Furosemide	25 mg, four times per day
Pantoprazole	20 mg, twice per day
Calcium carbonate	500 mg, four times per day
Calcitriol	0.25 µg, twice per day
Warfarin	Dosed to maintain an international normalized ratio between 2 and 3
Epoetin zeta	95 IU/kg/week

Approximately two weeks following the spontaneous resolution of respiratory symptoms and a negative SARS-CoV-2 nasopharyngeal swab result by reverse transcription-polymerase chain reaction, the patient presented to the emergency department (ED) in late April 2021 with intractable hiccups that had persisted for 48 hours. Upon admission, his vital signs were as follows: blood pressure, 180/105 mmHg; heart rate, 72 beats per minute; and oxygen saturation, 94%. The laboratory examination revealed a serum creatinine level of 3.2 mg/dL, blood urea nitrogen (BUN) of 78 mg/dL, hemoglobin of 12.1 mg/dL, and mild neutrophilia. The patient received an intravenous dose of 25 mg of chlorpromazine, which promptly induced significant sedation and demonstrated efficacy in alleviating the hiccups within a two-hour timeframe following administration. After six hours of additional observation in the ED, the patient was discharged home.

At the end of June 2021, the patient visited the nephrology department for his regular renal function monitoring. During the visit, the patient continued to suffer from violent hiccups, which he reported had persisted relentlessly for the past two months, both day and night. He mentioned that the relief provided by the chlorpromazine administered in the ED was ephemeral, and the hiccups recurred shortly after his discharge home. At that time, his renal function had further deteriorated, with a serum creatinine level of 4.1 mg/dL, a BUN of 91 mg/dL, and an estimated glomerular filtration rate (eGFR) of 15 mL/min, necessitating preparation for dialysis. The patient appeared prostrated by the intractable hiccups. Considering this and his limited renal function, the nephrologist advised the patient's general practitioner to attempt treatment with 10 mg of baclofen twice daily.

In late August 2021, approximately one month prior to the scheduled nephrologist outpatient visit, the patient revisited the ED due to his intractable hiccups. He reported that baclofen had failed to provide relief, and apart from a brief respite of a few hours following intravenous chlorpromazine administration, he had endured violent hiccups continuously from April 2021 to August 2021, a period of four months. At that point, his renal function had further deteriorated, with a serum creatinine level of 8.2 mg/dL, an eGFR of 6 mL/min, a BUN of 112 mg/dL, a hemoglobin level of 10.1 mg/dL, and a serum calcium level of 3.85 mg/dL. Due to the prostration caused by the intractable violent hiccups and poor general conditions, the patient was immediately hospitalized for clinical management and initiation of dialysis.

**Figure 1:**
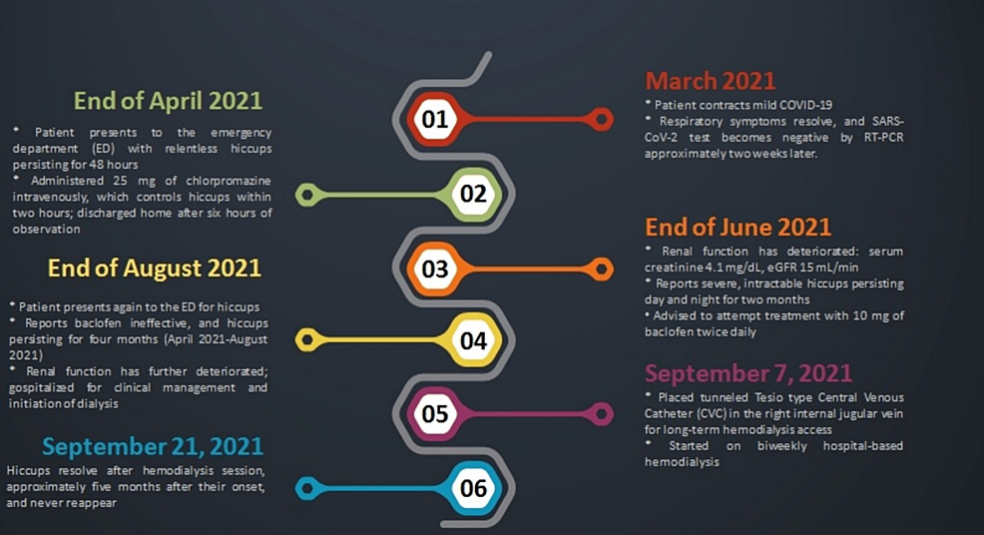
Timeline of the patient's history

Following the insertion of a tunneled Tesio-type central venous catheter into the right internal jugular vein for long-term hemodialysis access, the patient commenced biweekly hospital-based hemodialysis on September 7, 2021. At the time, he was still experiencing violent hiccups. However, after the hemodialysis session on September 21, 2021, approximately five months after their onset, the hiccups resolved and never reappeared. A detailed timeline of the patient's history is illustrated in Figure 1.

## Discussion

The most prominent aspect of our patient, who had a pre-existing diagnosis of stage IV CKD, was the sudden onset of intractable and violent hiccups two weeks following the resolution of a mild COVID-19 infection. These hiccups persisted continuously for a period of five months, with the sole exception being a brief period of remission lasting several hours after the administration of chlorpromazine. It is noteworthy that throughout the entire duration of the hiccups' onset and persistence, the patient's renal function exhibited a progressive deterioration, ultimately necessitating the urgent initiation of hemodialysis. The patient underwent their first biweekly hemodialysis session on September 7, 2021. Remarkably, following a hemodialysis session two weeks later, the patient experienced a complete resolution of the hiccups.

Several case reports have documented instances of persistent hiccups in patients with COVID-19 [7-10], often manifesting as an atypical neurological symptom [7] or resulting from inflammatory pneumonia-associated irritation of the phrenic nerve and its pericardial branch [8]. However, in our patient, we propose that the primary underlying mechanism was a SARS-CoV-2-induced acute deterioration of kidney function, superimposed on pre-existing chronic renal failure. This is supported by evidence that SARS-CoV-2 can infect kidney cells, including tubular epithelial cells and podocytes, potentially leading to acute tubular necrosis and interstitial inflammation [11]. Furthermore, the virus can cause endothelial injury and microvascular thrombi [12], which can further exacerbate kidney damage. The key evidence supporting the notion that intractable hiccups in our patient were primarily driven by uremia is the concurrent worsening of renal function parameters over the five-month period during which the violent hiccups persisted, followed by resolution after the initiation of hemodialysis. Our hypothesis is also consistent with previous observations that the accumulation of uremic toxins, coupled with electrolyte imbalances, may play a role in the development of hiccups [13]. However, the exact causative molecules and their specific point of action within the hiccup reflex arc remain unclear.

The clinical management of intractable hiccups can be particularly challenging for patients with advanced CKD. Generally, chlorpromazine is considered a first-line treatment option as it does not require renal dose adjustments, unlike other medications such as baclofen [13]. However, in our patient, chlorpromazine only provided temporary relief lasting a few hours. Despite the previously reported risk of baclofen-induced neurotoxicity in patients with chronic renal failure and intractable hiccups [14], a therapeutic attempt with baclofen was made in our patient but proved unsuccessful. Notably, only the initiation of hemodialysis resulted in the resolution of hiccups, underscoring the importance of addressing the underlying metabolic derangements in this complex clinical scenario. Our findings are entirely consistent with a previous case report by Nguyen et al. [13], who described a case of a patient with persistent hiccups associated with acute tubular injury and oxalate crystals, which resolved only with dialysis.

## Conclusions

This case highlights the challenges of managing intractable hiccups in patients with advanced CKD and underscores the importance of addressing underlying metabolic derangements in such complex clinical scenarios. The resolution of hiccups following hemodialysis initiation suggests a possible link between the hiccups and uremia and adds to the growing body of evidence supporting the role of dialysis in resolving intractable hiccups associated with severe renal dysfunction.

## References

[1] Steger M, Schneemann M, Fox M (2015). Systemic review: the pathogenesis and pharmacological treatment of hiccups. Aliment Pharmacol Ther.

[2] Reichenbach ZW, Piech GM, Malik Z (2020). Chronic hiccups. Curr Treat Options Gastroenterol.

[3] Kohse EK, Hollmann MW, Bardenheuer HJ, Kessler J (2017). Chronic hiccups: an underestimated problem. Anesth Analg.

[4] Polito NB, Fellows SE (2017). Pharmacologic interventions for intractable and persistent hiccups: a systematic review. J Emerg Med.

[5] Rouse S, Wodziak M (2018). Intractable hiccups. Curr Neurol Neurosci Rep.

[6] Rizzo C, Vitale C, Montagnini M (2014). Management of intractable hiccups: an illustrative case and review. Am J Hosp Palliat Care.

[7] Nakaya A, Ogura E, Katayama Y (2021). Hiccups as a specific neurological manifestation in males with COVID-19. IDCases.

[8] Ikitimur H, Borku Uysal B, Ikitimur B, Umihanic S, Smajic J, Jahic R, Olcay A (2021). Case report: two cases of persistent hiccups complicating COVID-19. Am J Trop Med Hyg.

[9] Totomoch-Serra A, Ibarra-Miramon CB, Manterola C (2021). Persistent hiccups as main COVID-19 symptom. Am J Med Sci.

[10] Giannos P, Katsikas Triantafyllidis K, Geropoulos G, Kechagias KS (2022). Persistent hiccups as an atypical presentation of SARS-CoV-2 infection: a systematic review of case reports. Front Neurol.

[11] He W, Liu X, Hu B (2022). Mechanisms of SARS-CoV-2 infection-induced kidney injury: a literature review. Front Cell Infect Microbiol.

[12] Canale MP, Menghini R, Martelli E, Federici M (2022). COVID-19-associated endothelial dysfunction and microvascular injury: from pathophysiology to clinical manifestations. Card Electrophysiol Clin.

[13] Nguyen V, Deeb K, Rathakrishnan R (2020). Hiccups: you got to be kidney me!. SAGE Open Med Case Rep.

[14] Chou CL, Chen CA, Lin SH, Huang HH (2006). Baclofen-induced neurotoxicity in chronic renal failure patients with intractable hiccups. South Med J.

